# Establishing the Carbonation Profile with Raman Spectroscopy: Effects of Fly Ash and Ground Granulated Blast Furnace Slag

**DOI:** 10.3390/ma14071798

**Published:** 2021-04-05

**Authors:** Yanfei Yue, Jing Jing Wang, P. A. Muhammed Basheer, Yun Bai

**Affiliations:** 1 College of Materials Science and Engineering, Chongqing University, 174 Shazheng Street, Shapingba, Chongqing 400044, China; 2 CRANN and AMBER Research Centers, Trinity College Dublin, D02 PN40 Dublin 2, Ireland; JJWANG@tcd.ie; 3 School of Civil Engineering, University of Leeds, Woodhouse Lane, Leeds LS2 9JT, UK; P.A.M.Basheer@leeds.ac.uk; 4 Department of Civil, Environmental and Geomatic Engineering, University College London, Gower Street, London WC1E 6BT, UK; yun.bai@ucl.ac.uk

**Keywords:** calcium carbonate, carbonation profile, fly ash, ground granulated blast furnace slag, Raman spectroscopy

## Abstract

Establishing the carbonation profile is of great significance to the prediction of the service life of reinforced concrete structures. In our previous work, Raman spectroscopy was shown to be an efficient tool for characterizing calcium carbonate (CaCO_3_) polymorphs and their profile in plain Portland cement (PC) matrices. However, as supplementary cementitious materials (SCMs), particularly fly ash (FA) and ground granulated blast furnace slag (GGBS), are widely used in concrete, establishing the carbonation profile without considering the possible effects of these SCMs could be of little significance to the real world. This paper, thus, investigated the effects of FA and GGBS on the working capacity and reliability of Raman spectroscopy for establishing the carbonation profile in PC blends containing SCMs. The thermogravimetry (TG) analysis was also conducted to verify the results from Raman spectroscopy. The results show that Raman spectroscopy demonstrated a good capacity for differentiating the variation of CaCO_3_ contents in FA or GGBS blends. However, the incorporation of FA and GGBS into the PC system caused some adverse effects on the quantification of CaCO_3_ by Raman spectroscopy, which could be attributed to the darker color and weak scatter nature of FA and the high content of glassy phases in GGBS.

## 1. Introduction

Concrete is inevitably exposed to carbonation attacks during its service life owing to the presence of carbon dioxide (CO_2_) such as that in the atmosphere. Briefly, carbonation occurs when the CO_2_ diffuses into concrete through its pore network, dissolves in the pore solution as CO_3_^2−^ ions and then reacts with various phases in the cement matrix [1]. Whilst virtually almost all of the Ca-bearing phases are susceptible to carbonation, calcium hydroxide (Ca(OH)_2_, CH, portlandite) has been commonly considered to be the phase that reacts most readily with CO_2_, leading to the formation of calcium carbonate (CaCO_3_) [1,2]. The consumption of CH in particular causes the drop of the pH in concrete, which subsequently triggers the depassivation of the rebar and ultimately leads to its corrosion in concrete [1,3]. In addition to the reaction with CH, CO_2_ can also attack calcium silicate hydrate (C-S-H), leading to the decalcification of the C-S-H gel as well as the formation of CaCO_3_ [4,5]. Evidently, the carbonation of concrete contributes significantly to the formation of CaCO_3_, which could exist in different polymorphs; namely, calcite, aragonite and vaterite [6]. Compared with the naturally occurring carbonation with a low CO_2_ concentration (around 0.03%) at a very slow rate, the accelerated carbonation (with a higher CO_2_ concentration), which is commonly adopted in laboratory evaluations, generally leads to the co-existence of all of these three polymorphs [7]. Furthermore, Ca-modified amorphous silica gel could also be formed from the carbonation of the C-S-H gel [7]. Although different products could be formed from the carbonation reaction, the most important phase that needs particular attention is the CaCO_3_ polymorph phase as this can cause significant changes to the properties of concrete. Specifically, whilst carbonation can enhance the mechanical properties of concrete through densifying the microstructure, the reduction in pH on the other hand can result in the corrosion of the reinforcing steel and hence the degradation of the performance of concrete. Therefore, accurate information about the carbonation in concrete will, no doubt, benefit the assessment of the durability performance of existing reinforced concrete (RC) structures [8,9]. 

In general, the amount of CaCO_3_ formed in carbonated concrete decreases with the increase of the distance from the exposed surface of concrete, which is mainly due to the reduced carbonation over the depth into concrete [5,10]. By plotting the CaCO_3_ content against the depth into concrete, a visual illustration of the distribution of CaCO_3_ inside concrete could be produced, forming the so-called ‘carbonation profile’ [5,6]. Establishing an accurate and reliable carbonation profile has been considered important not only because this can allow us to follow the carbonation front, but more importantly it can facilitate the prediction of the initiation of rebar corrosion and the service life of a reinforced concrete structure [11]. Additionally, as the formation of CaCO_3_ can also change a few other properties of concrete such as refining the pore structure as well as increasing the compressive and tensile strengths [12,13] a good knowledge of the distribution of CaCO_3_ may also benefit the understanding of the cause of these changes. Traditionally, the phenolphthalein spray test has been widely used to determine the carbonation depth by spraying the phenolphthalein indicator onto the freshly split surface of a concrete prism and taking a visual judgement of the color boundary [14]. Although this test can clearly indicate the fully carbonated region, it has been heavily criticized for its incapability of differentiating the uncarbonated and partially carbonated regions [15]. Moreover, the phenolphthalein test also cannot determine the content of CaCO_3_. Therefore, efforts have been made in the past to find better alternatives for establishing the carbonation profile. For example, the thermogravimetry (TG)/derivative thermogravimetry (DTG) analysis has been employed to establish CaCO_3_ content at different depths of specimens by tracing the DTG diagram related to CaCO_3_ decomposition [5,16]. However, this technique is less satisfactory in differentiating each CaCO_3_ polymorph, particularly the CaCO_3_ phase with a low thermal stability, as it decomposes in the near/same temperature range as CH. Although the X-ray diffraction (XRD) analysis could be used to establish the distribution of CaCO_3_, its sample preparation and data processing are complicated [17]. Other techniques such as the Gamma-ray attenuation method (GRAM)/Gammadensimetry and Fourier transformation infrared spectroscopy (FTIR) all suffer different limitations for quantifying different CaCO_3_ phases [6,14,15,16]. 

Raman spectroscopy, one of the most important vibrational spectroscopic analysis technologies, is an advanced analytical tool combining both the fingerprint identification capacity and quantification potential. As the wavelength (frequency) shift between the incident laser light and the Raman scattered light is specific to the chemical bond and the symmetry and vibrations of molecules, the Raman scattered signal (i.e., Raman shift) can thus provide ‘fingerprint’ information to recognize substances [18,19]. Due to its various advantages, researchers have attempted to employ Raman spectroscopy in the area of cement and concrete, e.g., for analyzing bottom ash fired bricks [20], as well as for the carbonation products formed in cementitious materials [21]. Although all of the calcium carbonate polymorphs, i.e., calcite, vaterite and aragonite, have been successfully characterized in previous research, no attempt has been made to quantify the calcium carbonate content in order to establish the carbonation profile. Besides, no research has been carried out on the Raman spectroscopic investigation of the carbonation in blended cement materials containing supplementary cementitious materials (SCMs) (e.g., fly ash (FA), ground granulated blast furnace slag (GGBS)), let alone establishing the carbonation profile in the blended cementitious matrices. In recent years, the authors have carried out extensive research to apply both bench-mounted Raman spectroscopy and the optical fiber Raman technique in cement and concrete, covering the different aspects related to cementitious materials such as the anhydrous phases and hydration products as well as the deteriorated phases/products [22,23,24,25,26]. It is worth highlighting that previous works have successfully developed bespoke optics-tailored fiber optic Raman systems for characterizing the carbonation products of concrete. In particular, the systems not only can qualitatively differentiate the polymorphs of calcium carbonates but can also quantitatively establish their profiles [23,26]. Nonetheless, previous studies were fully performed on plain (pure) Portland cement (PC) pastes without incorporating any SCMs. However, as SCMs, particularly FA and GGBS, are widely used in concrete practice to meet the increased requirements for durability and sustainability, establishing the carbonation profile without considering the possible effects of these SCMs on Raman spectra could be of little practical significance. This is, in particular, due to the fact that FA is an intrinsically weak Raman scatterer, which, coupled with its complex (e.g., iron oxide) and heterogeneous nature, has made it a difficult material to be analyzed by Raman spectroscopy [27]. GGBS on the other hand contains almost non-crystalline phases and has also been considered to be tricky to be analyzed by Raman spectroscopy probably due to its high percentage of disordered phases [27]. Indeed, intense fluorescence has been observed both in our own investigations and the studies reported in the literature when Raman spectroscopy has been employed to characterize FA or GGBS [27]. Depending on the intensity of the fluorescence, it could make a strong disturbance to or even totally hamper the weak Raman signals being obtained. Consequently, concerns could be raised over their potential effects on the quantification of CaCO_3_ when the Raman spectroscopy technique is used to establish the carbonation profile of PC blends containing FA or GGBS. 

The current work thus aims to investigate the working capacity of Raman spectroscopy for establishing the carbonation profile of FA or GGBS blended PC systems and more importantly to explore the potential effects that FA and GGBS may have on the working capacity and reliability of Raman spectroscopy. The CaCO_3_ polymorphs and the carbonation profile (i.e., the CaCO_3_ content against the depth) in the FA and GGBS blended cement pastes after being exposed to accelerated carbonation were characterized by a bench-mounted Raman spectroscope. The signal-to-noise ratios (SNR) of the Raman spectra collected from different cement systems were compared and the TG analysis was adopted as a supplementary tool to verify the Raman results and quantitatively assess the effects of FA and GGBS on the carbonation profile established based on the content of CaCO_3_. It is anticipated that the results obtained from this study will lay a foundation for analyzing the similar effects that FA and GGBS may have on the optical fiber Raman system in the future.

## 2. Materials and Methods

### 2.1. Materials

**Figure 1:**
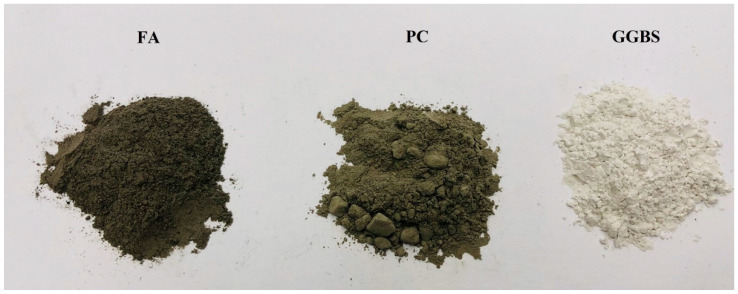
Photo of fly ash (FA), Portland cement (PC) and ground granulated blast furnace slag (GGBS).

In this study, Portland cement (PC) CEM I 42.5N supplied by Quinn Cement (Derrylin, County Fermanagh, Northern Ireland, UK) (BS EN 197-1:2011) was used to formulate blended cementitious systems. The low calcium fly ash (FA), conforming to the Category S according to BS EN 450-1:2012, was supplied by Connexpo (N.I.) Ltd. from Kilroot Power Station in Antrim Northern Ireland, UK. The specific surface area and specific density of the FA was 340 m^2^/kg and 2520 kg/m^3^, respectively. The ground granulated blast furnace slag (GGBS) was supplied by Civil and Marine Ltd., Maidenhead, UK. The GGBS was an off-white color powder with a fineness of 527 m^2^/kg and a loose bulk density of 1000–1100 kg/m^3^. The photo indicating the color of the FA, PC and GGBS is shown in Figure 1 and their chemical compositions are presented in Table 1. A Sikaguard 680-s acrylic paint supplied by Sika Switzerland was employed to coat the paste samples before carbonation (as detailed in Section 2.3).

### 2.2. Manufacture of FA/PC and GGBS/PC Blends

The FA/PC and GGBS/PC pastes were manufactured with a water/binder ratio of 0.5, which enabled both mixes to achieve a similar workability in terms of the mini-slump value in the range of 85 ± 5 mm. The percentage of FA and GGBS in the cement blends was 35% and 50%, respectively. These are the typical cement replacement levels for FA and GGBS, respectively, in practice. Prior to mixing, the PC and FA or GGBS powders were placed into a plastic container and mixed by rotating the container by hand for three minutes in an attempt to achieve a thorough mixing of the powders. The pastes were then manufactured by mixing the water and FA/PC or GGBS/PC powder using a Hobart planetary mixer following the procedures specified in BS EN 196-3:2005+A1. To avoid any variation between batches, the specimens needed for each mix (i.e., FA/PC and GGBS/PC) were cast in one batch. The obtained pastes were grey color slurries with appropriate flowability (i.e., a mini-slump value of 85 ± 5 mm as aforementioned).

Immediately after mixing, the cement pastes were cast in two layers into cylindrical PVC molds (size of Ø50 mm × 80 mm) with each layer being vibrated for several seconds to remove the entrapped air. Following this, the specimen surfaces were finished with a steel scraper. The molds (with specimens) were then sealed with lids and cured in a constant-temperature room at 20 (± 1) °C for 24 h.

### 2.3. Curing, Conditioning and Carbonation

After 24 h initial curing in the molds, the specimens were demolded and wrapped individually with a water-saturated hessian. The hessian was regularly checked and wetted to ensure the samples were fully saturated. The specimens were then sealed in plastic bags and stored again in the constant-temperature room at 20 (± 1) °C for 55 days.

After a total of 56 days’ curing, the specimens were dried in an oven at 40 (± 1) °C for 14 days. The specimens were then wrapped individually with a polythene sheet and placed again in the 40 (± 1) °C oven for another 14 days to redistribute the moisture and achieve an internal relative humidity (RH) of about 60 (± 5)% for the subsequent accelerated carbonation. Following this, the specimens were cooled down to 20 (± 1) °C for one day and all faces, except the face cast against the molds, were coated with three layers of Sika acrylic paint. After being dried under 20 (± 1) °C for five days, the painted specimens were stored in a carbonation chamber (LEEC, UK) at a constant temperature of 20 (± 1) °C with a CO_2_ concentration of 5 (± 0.5)% and RH of 60 (± 5)% for six weeks. 

**Figure 2:**
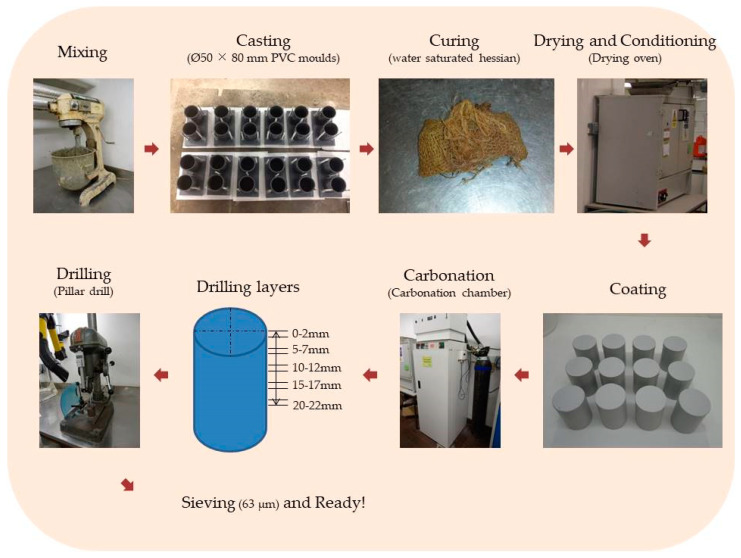
Flowchart illustrating the sample preparation procedure.

After the carbonation, the powder samples were collected at five discrete depths from the exposed surface inwards (namely, 0–2 mm, 5–7 mm, 10–12 mm, 15–17 mm and 20–22 mm) of the specimens using a digital drill (with an accuracy of ± 0.1 mm) with a bit of 8 mm diameter and 4 mm deep slant edge. The collected powders were then passed through a 63 µm sieve. To avoid any contamination to the samples, a vacuum cleaner was employed to suck away the powders from the intervals (i.e., 2–5 mm, 7–10 mm, 12–15 mm and 17–20 mm). The obtained powder samples (i.e., the particles below 63 µm) were then put into airtight plastic bags and stored in a vacuum desiccator until being analyzed by Raman and TG tests. Figure 2 illustrates the sample preparation procedure involved during the experiments.

### 2.4. Bench-Mounted Raman Spectroscopy

A bench-mounted Renishaw Raman microscope with a charged coupled device (CCD) (Renishaw, Gloucestershire, UK) was used in this study, which worked under a controlled temperature of 20 °C. The laser was a 514.5 nm single-line (Argon ions) laser and its beam was focused into the sample through a LEICA (Wetzlar, Germany) N Plan objective (10×). The measured power at the sampling level was around 3.2 mW. Before each experiment, the Raman shift was calibrated using the sharp peak of the TiO_2_ (anatase) powder. The analyses were performed with an exposure time of 10 s and accumulations of 10 to achieve an appropriate signal-to-noise ratio (SNR).

### 2.5. Background Subtraction

During the data process, the background of the Raman spectra was subtracted by adopting the baseline correction using Origin 2018 (OriginLab, Northampton, MA, USA). This procedure can remove the strong fluorescence background, allowing the Raman peaks to be clearly observed.

### 2.6. Calculation of the Signal-to-Noise Ratio (SNR)

The SNR values of the Raman spectra were calculated using the υ_1_ CO_3_ peak of the samples obtained at the depth of 0–2 mm according to the method recommended in American Society for Testing Materials (ASTM ) E579-04 as follows:

SNR = Signal level/Noise (RMS) level(1)

where the signal level is the peak height/intensity after subtracting the background and the noise level is obtained by the root mean square (RMS) method, which is the standard deviation of the intensity values of a selected Raman shift region on the spectrum after subtracting the background.

### 2.7. Thermogravimetry (TG)

To verify the results obtained from the Raman analysis, the well-established TG analysis was employed to assess the CaCO_3_ quantity. A NETZSCH STA 449C instrument (NETZSCH, Selb, Germany), which worked under an inert environment (N_2_) with a flow rate of 1 mL/min, was used. The samples were placed in an alumina (Al_2_O_3_) crucible and heated from room temperature to 1000 °C at a heating rate of 20 °C/min. The derivative thermogravimetric curves (DTG) were also recorded simultaneously. The CaCO_3_ quantity was calculated by following its decomposition to CaO and CO_2_ at a temperature of around 470 °C~820 °C.

## 3. Results and Discussion

To identify the possible effects that FA or GGBS might impose on the qualitative and, particularly, the quantitative working capacity of Raman spectroscopy for establishing carbonation profiles in blended cementitious systems, the experiment work was organized into two parts and the results are reported accordingly in this section as follows:

1. Establish the carbonation profiles viz. the variation of CaCO_3_ content against the depth of FA or GGBS containing PC pastes after being subjected to an accelerated carbonation with bench-mounted Raman spectroscopy.
2. Verify the carbonation profiles as developed by Raman spectroscopy at stage 1 by the well-established TG analysis so that the potential effects that FA or GGBS may have on the reliability of the results obtained from Raman spectroscopy could be recognized.

### 3.1. Establishing the Carbonation Profiles of FA/PC and GGBS/PC Pastes with Bench-Mounted Raman Spectroscopy

#### 3.1.1. Raman Spectroscopy Analysis

**Figure 3:**
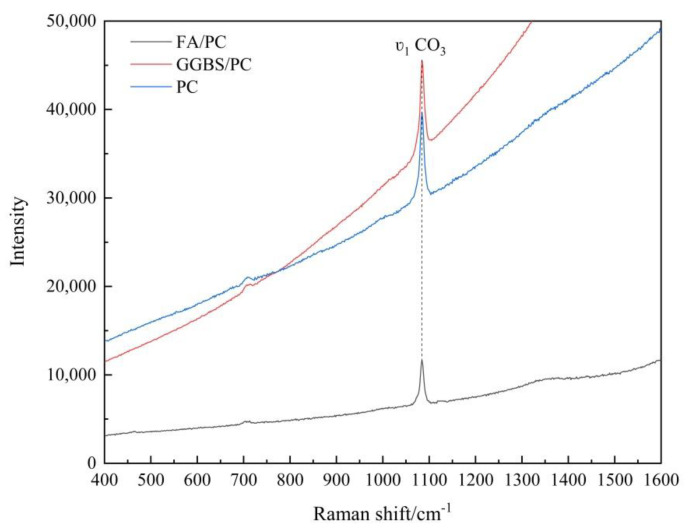
Raman spectra of the powders collected at a depth of 0–2 mm of the carbonated FA/PC, GGBS/PC and PC paste samples.

As aforementioned, FA is an intrinsically weak Raman scatterer whilst GGBS is also difficult to analyze by Raman spectroscopy due to its highly disordered amorphous phases. As exemplified in Figure 3 by the Raman spectra of the carbonated FA/PC, GGBS/PC and pure PC pastes at the depth of 0–2 mm, which all clearly showed an intense sharp peak at about 1085 cm^−1^ due to the υ_1_ (i.e., symmetrical stretching) vibration of CO_3_ of the CaCO_3_ phases [28,29,30,31], the intensity of the υ_1_ CO_3_ band of the FA/PC blend was much weaker (only 51% of that of the pure PC paste, as shown in Table 2) than those of both GGBS/PC and pure PC. In addition to the weak Raman scatter nature of FA, the weak intensity from the FA/PC blend could also be attributed to the dark color of FA (as shown and compared with PC and GGBS in Figure 1). This is because, as well-established, the phases with the dark color can absorb more illuminating light energy [32]. As a result, the scattered light intensity could be reduced due to the reduced incident light energy. This could partly explain the much reduced Raman intensity of the FA/PC blend in Figure 3. Although the intensity of the υ_1_ CO_3_ band of GGBS/PC was not reduced (104% of PC) compared with that of PC, a stronger fluorescence background could be recognized. As the peak height is normally used to quantify the phases in a Raman analysis (as detailed in Section 3.1.2), the interferences caused by FA to the intensity and by GGBS to the fluorescence background of the Raman spectra in Figure 3 could cause concerns about the quantification of CaCO_3_ formed in the different mixes before establishing the carbonation profiles. To quantitatively identify the possible effects that FA and GGBS could have imposed on the quality of the Raman spectra, the SNR values of the Raman spectra in Figure 3 were calculated using the υ_1_ CO_3_ band according to the method described in Section 2.6 and the results are reported in Table 2. As can be seen from Table 2, under the Raman spectroscopy analysis, the SNR of the spectra obtained from the GGBS/PC paste was lower than that of PC, i.e., 75 vs. 81, while an even lower SNR (47) was obtained from the FA/PC paste, which could be primarily due to the much reduced signal level (51% of PC) of the FA/PC paste. On the other hand, the relatively lower SNR from GGBS/PC compared with the pure PC mix could be mainly caused by the higher noise level of GGBS (113% of PC), which is presumably due to the highly disordered amorphous phases in GGBS. Nonetheless, a strong background could be identified from all the three Raman spectra and in particular the GGBS/PC blend and the pure PC samples. As both PC and GGBS were subjected to a grinding treatment during their manufacture, the higher background from PC and GGBS could also be caused by the grinding agent as well as the grinding itself because grinding could also introduce different defects, promoting the formation of a strong fluorescence background [33]. Therefore, before CaCO_3_ could be quantified, the interference from the strong fluorescence background has to be removed first. This could also facilitate the identification of the possible effects that FA and GGBS could have introduced to the carbonation profile to be established in the next section. The background of the spectra collected at the different depths was therefore subtracted by following the procedures described in Section 2.5 and the details are presented below.

**Figure 4:**
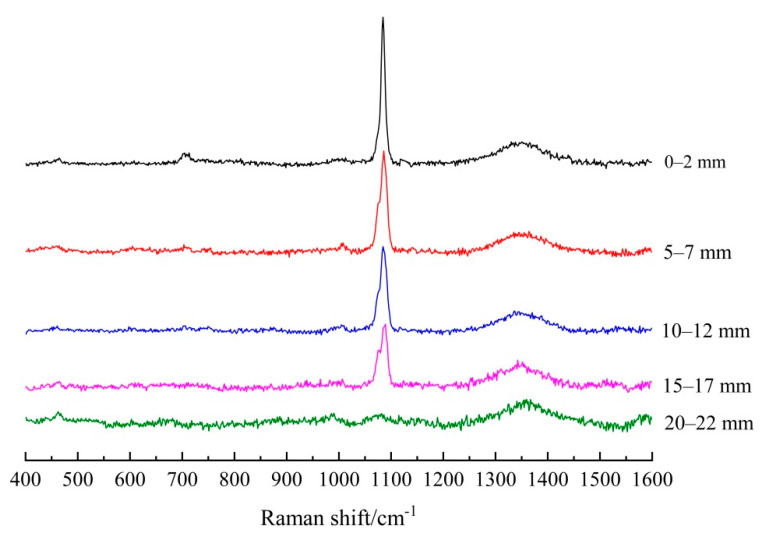
Raman spectra (after subtracting the background) of the powders collected at five depths of carbonated FA/PC paste samples.

**Figure 5:**
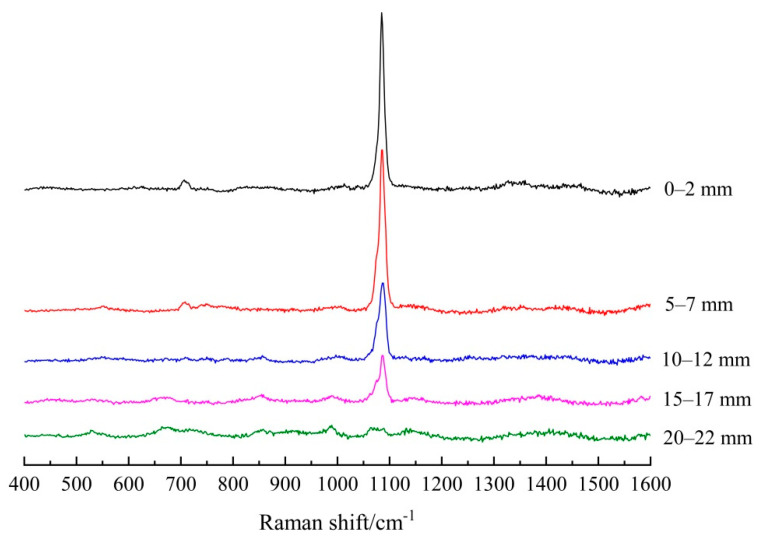
Raman spectra (after the subtracting background) of the powders collected at five depths of carbonated GGBS/PC paste samples.

Figure 4 and Figure 5 present the Raman spectra after subtracting the background from five different depths of the carbonated FA/PC and GGBS/PC paste cylinders, respectively. As shown in Figure 4, for the FA/PC blend, there were sharp peaks located at about 1085 cm^−1^, 1075 cm^−1^ and 1091 cm^−1^ (i.e., υ_1_ symmetrical stretching bands of CO_3_) being identified at 0–2 mm, 5–7 mm, 10–12 mm and 15–17 mm, indicating the formation of different CaCO_3_ polymorphs (viz. calcite, aragonite, vaterite) at these depths [28,29,30,31]. As highlighted before, the co-existence of these three CaCO_3_ polymorphs could be possible in the current study due to the severe carbonation condition applied, i.e., 5% CO_2_, during the accelerated carbonation test. In addition, the in-plane bending mode of the CO_3_ (υ_4_ CO_3_) band of calcite/aragonite was also identified at the two outermost depths, viz. 0–2 mm and 5–7 mm, as could be observed at about 707 cm^−1^ [28,29,30,31]. The weak band that emerged at about 1349 cm^−1^ could not be assigned appropriately, which could be attributed to a few impurities in the raw cement. In Figure 5, the υ_1_ CO_3_ peaks of the CaCO_3_ polymorphs at the first four depths were also clearly identified by Raman spectroscopy in the GGBS/PC blend as shown at 1085 cm^−1^ (calcite/aragonite) and 1075 cm^−1^/1090 cm^−1^ (vaterite). Additionally, the υ_4_ CO_3_ band of calcite/aragonite was identified at the outmost two depths (i.e., 0–2 mm and 5–7 mm), as indicated at about 707 cm^−1^ [28,29,30,31]. Most importantly, as clearly shown in both Figure 4 and Figure 5, the intensity of the υ_1_ CO_3_ band decreased with the increase of the depth, indicating the reduced amount of CaCO_3_ formed over the depth into the hardened PC blends of both systems, which could be mainly attributed to the reduced penetration of the CO_2_ into the FA and GGBS bearing PC pastes, respectively, under the accelerated carbonation.

#### 3.1.2. Establishing the Carbonation Profiles

In the previous section, a decreasing trend of the υ_1_ CO_3_ band over the depth in both the FA/PC and GGBS/PC pastes was clearly recognized. To identify the feasibility of establishing the carbonation profiles (in terms of the variation of the CaCO_3_ content against depth) using Raman spectroscopy, the peak heights (acting as the indicator of CaCO_3_ quantity) of the υ_1_ CO_3_ band at the five depths of the FA/PC and GGBS/PC pastes were retrieved and are presented in Table 3. In addition, the peak heights of the pure PC samples are also presented for comparison purposes [26]. In Table 3, it can be noticed that the carbonation fronts of the FA and GGBS containing pastes were deeper (until 15–17 mm) than that of the PC system (until 10–12 mm), as no CaCO_3_ was identified at the depth of 15–17 mm onwards in the PC pastes. This phenomenon is consistent with the literature showing that the addition of the mineral additions such as FA and GGBS could reduce the resistance to carbonation attack [34,35]. Additionally, for all of the three systems, CaCO_3_ was not identified at the depth of 20–22 mm, indicating these most inner regions might have not been carbonated.

**Figure 6:**
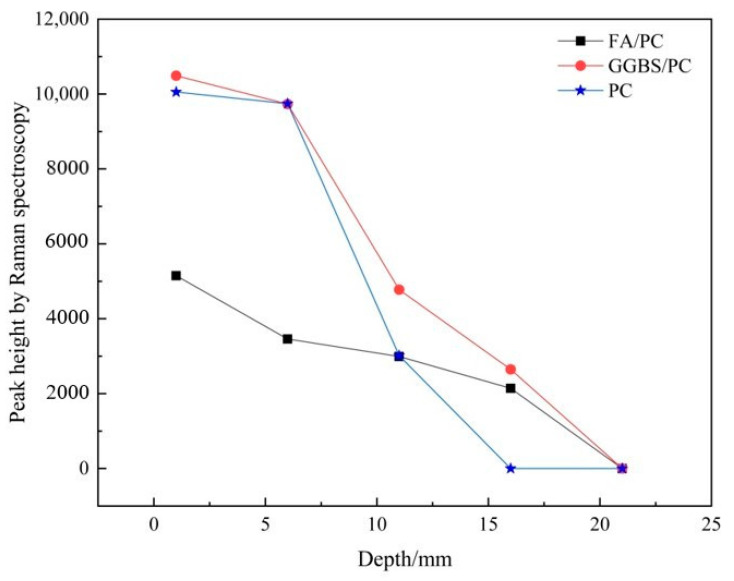
Relationship between the depth and peak height obtained from carbonated FA/PC, GGBS/PC and PC paste samples under Raman spectroscopy.

To develop a more visual illustration of the carbonation profiles, the peak height was then plotted against the depth as shown in Figure 6. Evidently, for both the FA/PC and GGBS/PC blends, there was an inverse relationship between the height (intensity) of the υ_1_ CO_3_ peak and the depth, indicating the decrease of the CaCO_3_ content with the increase of the sampling depth. Obviously, these two profiles displayed very similar patterns to that of the PC paste, i.e., the peak height of all of the three cementitious materials, viz. FA/PC, GGBS/PC and pure PC pastes, showed a declining trend with the increasing depth. This phenomenon correlated well with the typical carbonation profile, i.e., the CaCO_3_ content decreased with the depth. However, the peak intensities of the FA/PC blends at the depths of 0–2 mm and 5–7 mm were much lower than that of the GGBS/PC and PC pastes. Although a lower CaCO_3_ content from the FA/PC mix was somehow expected due to its reduced calcium bearing phases, the reduced incident laser power for the Raman scattering of the FA/PC mix owing to the darker color of FA could also have contributed to this phenomenon, which will be further discussed in Section 3.2.2 below. 

Therefore, based on the above results and discussion, it can be seen that introducing FA/GGBS into the pure PC mix has indeed caused some effects on the Raman spectra, which could be mainly attributed to the dark color and weak scatter nature of FA and the highly disordered phases in GGBS. However, although there were differences in the background, noise level and signal intensity between the Raman spectra obtained from the different cementitious systems, the qualitative working capacity of Raman spectroscopy for recognizing different CaCO_3_ polymorphs was not affected. Nonetheless, due to the differences in the peak heights between the different cementitious systems, the quantitative working capacity of Raman spectroscopy might have been affected and this was to be identified through the TG verification and is described in the next section below.

### 3.2. Verifying the Carbonation Profiles by TG Analysis

In the above section, using the peak height of the υ_1_ CO_3_ band as the indicator of CaCO_3_ content, the carbonation profiles of the FA/PC and GGBS/PC blends were established by Raman spectroscopy. However, due to the effects of FA and GGBS, the reliability of the quantified information as obtained from the Raman spectroscopy technique was still uncertain. In this section, derivative TG analysis (i.e., DTG) was employed as a well-established quantitative technique to verify the quantitative information and, hence, the carbonation profiles established by Raman spectroscopy.

#### 3.2.1. Thermogravimetry (TG) Analysis

**Figure 7:**
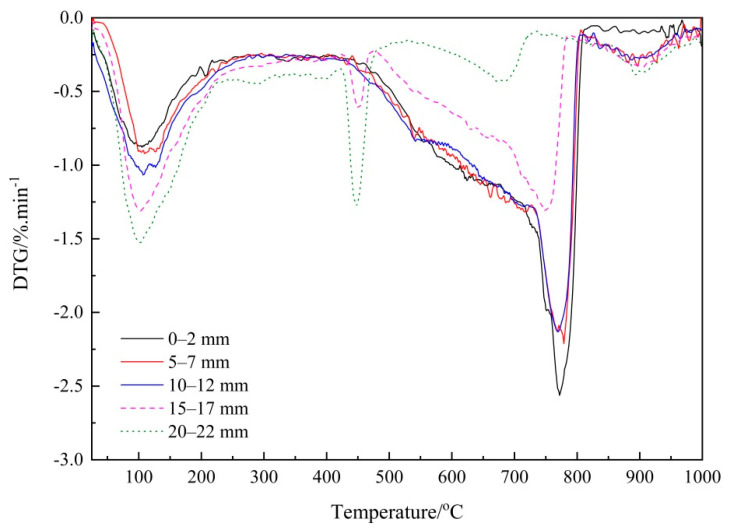
Derivative thermogravimetric (DTG) curves of the carbonated FA/PC paste samples at five depths.

**Figure 8:**
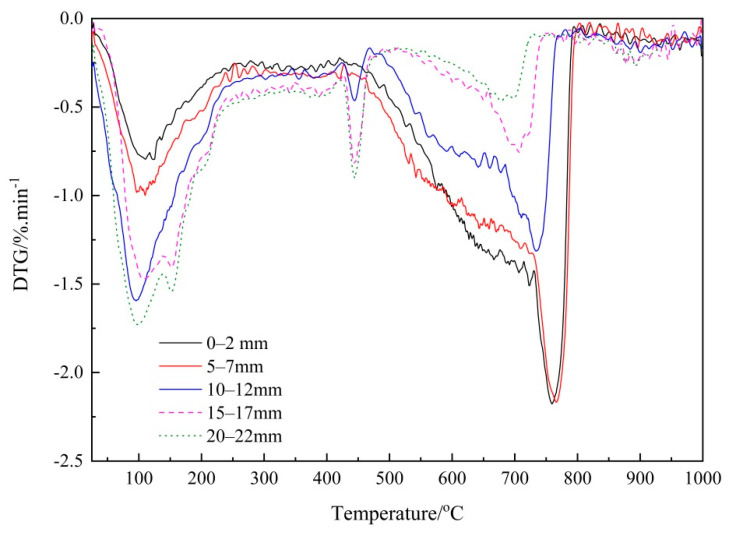
DTG curves of the carbonated GGBS/PC paste samples at five depths.

To clearly identify each individual phase, the DTG curves of the FA and GGBS blended cement pastes are presented in Figure 7 and Figure 8, respectively. As shown in Figure 7, the decomposition of CaCO_3_ in the FA/PC paste occurred at the temperature range about 470 °C~820 °C, with a peak centered at about 780 °C. Based on the literature, this could suggest the co-existence of the three CaCO_3_ polymorphs; namely, calcite, aragonite and vaterite [5]. An endothermic peak emerged at about 475 °C, which could be due to the dehydration of the portlandite in the cementitious materials. However, this peak disappeared from the first three depths of the FA blended paste, indicating that there was almost no portlandite existing in the first three depths of the FA blends, which could have been consumed by the reactions with CO_2_ (carbonation) or siliceous/alumino-siliceous in FA (pozzolanic reaction). On the other hand, in Figure 8, the endothermic peak for CaCO_3_ in the GGBS/PC samples emerged at about 760 °C with a related decomposition temperature ranging from around 470 °C to 810 °C, which corroborated the sharp mass loss due to the decomposition of the CaCO_3_ phases. Meanwhile, the decomposition temperature for portlandite was also observed at about 420 °C~470 °C with its peak centered at about 450 °C. These peaks became more evident in the deeper depths, i.e., 10–12 mm, 15–17 mm and 20–22 mm, indicating that a considerable amount of portlandite still existed in these areas due to reduced carbonation.

Moreover, in both Figure 7 and Figure 8 it is apparent that the integrated area of CaCO_3_ during the temperature range, which was linked to the quantity of CaCO_3_, decreased with the sampling depth, implying the possible decrease of the CaCO_3_ content. To exactly quantify the variation of the CaCO_3_ content, the area of the DTG curve at the decomposition temperature range of CaCO_3_ was determined accordingly and then used to calculate the content of CaCO_3_. Table 4 reports the obtained CaCO_3_ content and those of the PC paste samples are again listed for comparison purposes [26]. As can be seen from Table 4, considerable amounts (>8%) of CaCO_3_ were identified at the first four depths of the FA blended paste cylinders while only small quantity of CaCO_3_ was identified at the depth of 20–22 mm. For the GGBS/PC blends, the CaCO_3_ contents within the outermost three depths (0–2 mm, 5–7 mm and 10–12 mm) were relatively high (>9%), but decreased from a depth of 15–17 mm. These results, overall, correlate well with the Raman spectroscopy results (Table 3) in which CaCO_3_ was characterized at the first four depths of the samples. However, as small amounts of CaCO_3_ were identified by TG at the innermost depth (20–22 mm) of the carbonated paste cylinders, but not detected by Raman spectroscopy, it seems that further research is still needed to investigate how to improve the sensitivity of Raman spectroscopy.

#### 3.2.2. Verifying the Carbonation Profiles

**Figure 9:**
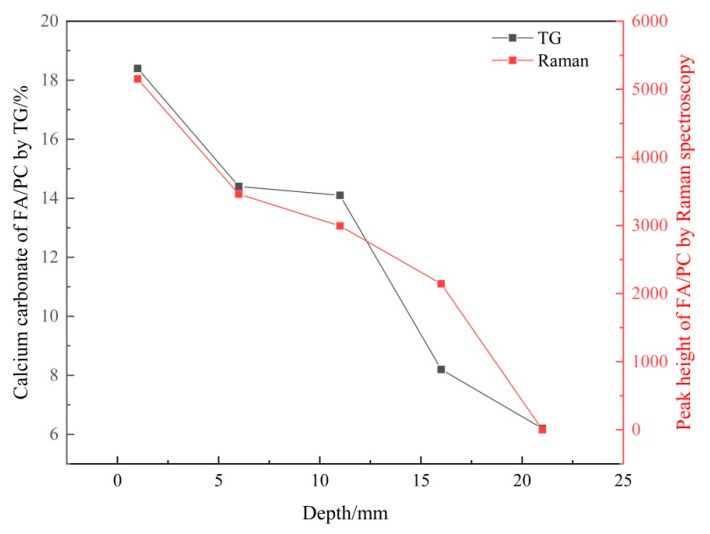
Carbonation profiles of the carbonated FA/PC paste samples established by Raman spectroscopy and TG analyses.

**Figure 10:**
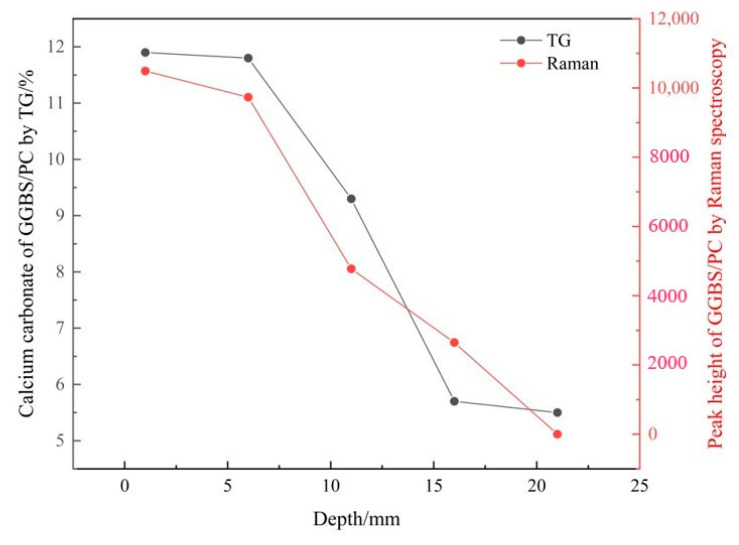
Carbonation profiles of the carbonated GGBS/PC paste samples established by Raman spectroscopy and TG analyses.

Based on the CaCO_3_ content obtained from the TG analysis (shown in Table 4), the carbonation profiles of the FA/PC and GGBS/PC pastes established by Raman spectroscopy using the peak height of υ_1_ CO_3_ as an indicator of the CaCO_3_ content were plotted together with the counterpart obtained from the TG analysis as seen in Figure 9 and Figure 10 respectively. Apparently, for both the FA and GGBS blended cement pastes, the carbonation profiles obtained from these two techniques (i.e., Raman spectroscopy and TG), both of which showed a reducing trend, were highly comparable. As TG is a well-established technique for quantifying CaCO_3_ in cementitious materials, the above results demonstrated a reasonable working capacity of the Raman spectroscopy for differentiating the variation (i.e., the relative quantity) of the CaCO_3_ content in carbonated FA/PC or GGBS/PC blends over the depth. However, from Figure 9 and Figure 10, it was not clear whether the CaCO_3_ content established from Raman spectroscopy could be used as an indicator of the real quantity (i.e., the absolute quantity) of CaCO_3_. This will be discussed in the sections below.

**Figure 11:**
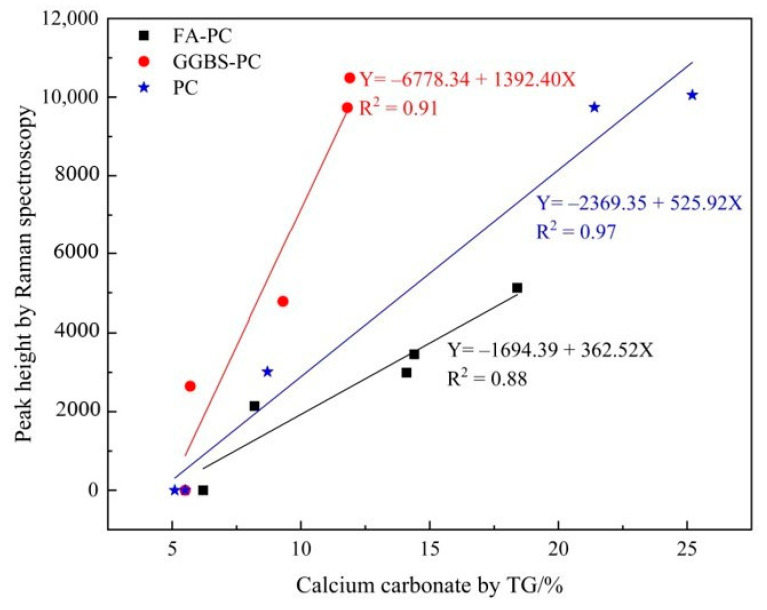
Correlation between the calcium carbonate in the carbonated FA/PC, GGBS/PC and PC paste samples identified by Raman spectroscopy and TG analyses.

To quantitatively and, hence, fully verify the working capacity of Raman spectroscopy for developing carbonation profiles, the correlations between the Raman and TG analyses were established and compared in Figure 11. As can be seen from Figure 11, for the FA/PC and GGBS/PC blends, there were reasonably good correlations between the contents of CaCO_3_ obtained from TG and the peak of the Raman spectroscopy analysis as demonstrated by their correlation coefficient (R^2^) values, i.e., 0.88 for FA/PC and 0.91 for the GGBS/PC system. Nonetheless, there was no doubt that the addition of FA and GGBS into the PC system clearly reduced these correlations as exemplified by the relatively lower R^2^ values of the FA/PC (0.88) and the GGBS/PC (0.91) blends compared with that of the pure PC (0.97). This indicated that FA and GGBS could cause some adverse effects on the quantification of CaCO_3_ by Raman spectroscopy with FA demonstrating more negative effects than GGBS. As previously discussed, the relatively darker color of FA could absorb more energy from the incident laser light leading to reduced Raman scattering and, hence, a reduced peak intensity. This, along with the weak Raman scatter nature of FA, could potentially reduce the intensity of the Raman spectrum even if the same amount of CaCO_3_ might have existed (as clearly demonstrated by the lower peak intensity reported in Table 2). Additionally, it should be highlighted that, as clearly demonstrated in Figure 1, the darkness of the raw materials increased in the order GGBS < PC < FA. Accordingly, one may expect that the consumption of light energy by the raw materials should also be in the same order of GGBS < PC < FA, which clearly coincided well with the peak intensities in Figure 11 even though the exact nature that caused the difference in intensity still needs further research. Additionally, the reduced correlation from the GGBS/PC blend could also be attributed to the possible interference from the increased noise level. As a result, as demonstrated in Figure 11, if the peak intensity was used to compare the quantity of CaCO_3_ across different cementitious systems, there was a potential risk that CaCO_3_ in the FA/PC blend could be underestimated whilst that in the GGBS/PC could be overestimated. The above results would, therefore, indicate that simply using the peak intensity as a quantity indicator might not be able to provide reliable information on the quantity of CaCO_3_ due to the effects of FA and GGBS. To overcome these effects, a quantified Raman analysis with an internal standard [36,37] similar to quantitative X-ray diffraction (QXRD) might need to be considered in future studies. Alternatively, if the Raman spectroscopy system is to be developed into a future monitoring system for the continued monitoring of the durability of concrete structures, individual calibration curves could be established for each type of concrete.

## 4. Conclusions

Carbonation initiates the corrosion of reinforcing steel and alters the mineralogical composition as well as the microstructure of the cementitious materials. Thus, characterizing the CaCO_3_ polymorphs and in particular establishing the carbonation profiles are essential for predicting the service life and the health condition of concrete structures. The current work employed Raman spectroscopy to establish the carbonation profiles (in terms of the variation of CaCO_3_ content against the depth) of FA and GGBS blended cementitious materials. Additionally, the potential effects that FA and GGBS might impose on the quantitative working capacity of Raman spectroscopy was systematically investigated. From the results obtained in this study, it showed that Raman spectroscopy was adequate to differentiate the variation (i.e., the relative quantity) of the CaCO_3_ content and hence there is a potential that the carbonation profiles of the FA/PC or GGBS/PC blended cementitious materials can be established with the Raman spectroscopy technique. However, the addition of FA and GGBS clearly reduced the correlations between the contents of CaCO_3_ obtained from TG and the peak intensity of the Raman spectroscopy analysis as exemplified by the lower R^2^ values obtained. Therefore, using the peak intensity as the indicator of the CaCO_3_ content, the CaCO_3_ content in the FA/PC blend could be underestimated whilst that in the GGBS/PC could be overestimated. These adverse effects could be due to the darker color and weak scatter nature of FA and the high content of glassy phases in GGBS, respectively. To solve this problem, a quantified Raman analysis with an internal standard or individual calibration curve has to be employed before this technique can be used as a reliable alternative approach to monitoring the carbonation attack in concrete structures.

## Figures and Tables

**Table 1:** Chemical composition of the PC, FA and GGBS.

Oxides/%	SiO_2_	Al_2_O_3_	Fe_2_O_3_	CaO	MgO	K_2_O	Na_2_O	SO_3_
PC	23.00	6.15	2.95	61.30	1.80	0.68	0.22	2.50
FA	56.30	23.50	4.70	4.40	1.80	1.80	1.00	0.90
GGBS	34.30	15.00	0.40	39.40	8.00	0.38	0.45	0.80

**Table 2:** Summary of the signal, noise and signal-to-noise ratio (SNR) of the Raman spectra of the powders collected at a depth of 0–2 mm of the carbonated FA/PC, GGBS/PC and PC paste samples.

Parameters of Spectra	FA/PC Paste	GGBS/PC Paste	PC Paste
Signal (peak intensity)	5151.5 (51%)	10,491.3 (104%)	10,055.1 (100%)
Noise	110.0 (89%)	139.8 (113%)	124.2 (100%)
SNR	47	75	81

Note: the signal and noise were normalized based on that of the PC paste and shown in brackets.

**Table 3:** Summary of the calcium carbonate content (in terms of peak height) at five depths of carbonated FA/PC, GGBS/PC and PC paste samples under Raman spectroscopy.

Depth/mm	FA/PC Paste	GGBS/PC Paste	PC Paste
0–2	5151.5	10491.3	10,055.1
5–7	3461.5	9734.5	9741.7
10–12	2993.9	4776.6	3014.8
15–17	2143.7	2650.0	0
20–22	0	0	0

**Table 4:** Summary of the calcium carbonate content (%) at five depths of carbonated FA/PC, GGBS/PC and PC paste samples under a thermogravimetry (TG) analysis.

Depth/mm	FA/PC Paste	GGBS/PC Paste	PC Paste
0–2	18.4	11.9	25.2
5–7	14.4	11.8	21.4
10–12	14.1	9.3	8.7
15–17	8.2	5.7	5.5
20–22	6.2	5.5	5.1

## Data Availability

Data are contained within the article.
